# 2959. Urinary tract colonizing abilities of fosfomycin (FOF) non-susceptible *Klebsiella pneumoniae* (KP) inner colonies (IC) arising from disk diffusion (DD) in an ascending mouse urinary tract infection (UTI) model

**DOI:** 10.1093/ofid/ofad500.198

**Published:** 2023-11-27

**Authors:** Morgan Bixby, Lindsey Collins, Jenna Salay, Connie Clabots, Elizabeth B Hirsch

**Affiliations:** University of Minnesota College of Pharmacy, Minneapolis, MN; University of Minnesota College of Pharmacy, Minneapolis, MN; University of Minnesota College of Pharmacy, Minneapolis, MN; Minneapolis VA Medical Center, Minneapolis, MN; University of Minnesota College of Pharmacy, Minneapolis, MN

## Abstract

**Background:**

DD is a common method for FOF susceptibility testing against Enterobacterales, despite a lack of breakpoints for non-*E. coli* organisms. When extrapolating these breakpoints, methods for reading zone diameters from the breakpoint setting organizations are often extrapolated as well. With CLSI and EUCAST in disagreement on interpretation of IC that arise from DD, it is imperative that the clinical relevance of these IC is determined. We sought to determine the urinary tract colonizing ability of one clinical KP isolate via an ascending mouse UTI model.

**Methods:**

The parent isolate, KP131—a clinical isolate from Boston, MA—had an MIC of 8 µg/mL and its IC from DD had an MIC of 1024 µg/mL. Immune competent female CBA/j mice (n = 35) were inoculated with an equal mix of KP 131 parent/IC culture via urinary catheter at 1µL/g mouse weight, which delivered a total of 10^11^ CFU/mL per mouse bladder. At 48 hours post-inoculation, mice were sacrificed, and kidneys/bladders were aseptically collected and individually homogenized. Homogenate was plated on both standard Mueller-Hinton agar (MHA) and MHA plates containing 32 µg/mL FOF + 25µg/mL glucose-6-phosphate. The number of parent colonies were determined by subtracting the FOF-supplemented plate counts from the standard MHA counts and proportions compared via Kruskal-Wallis test.

**Results:**

Of 35 mice infected, 14.3% (n = 5) died prior to harvest. Bladder colonization occurred in 80% (n/N = 24/30) of mice and in one or both kidneys in 67% (n/N = 16/24) of mice when bladders were colonized; 25 total kidneys were colonized. Among these, IC accounted for 44.8% of bacterial burden in the bladder and 54.6% in the kidneys. There was no significant difference in the colonizing abilities of IC compared to the parents in the bladder (p = 0.289). However, the IC colonized at a significantly higher rate (p = 0.046) than the parent isolate in the kidney tissue.

**Conclusion:**

Results from this preliminary study show that KP IC have the ability to colonize the urinary tract at near equal rates to the parent culture when in competition in a mouse UTI model, including a significantly greater colonization in kidney tissue. While this work is limited by use of a single parent/IC pair, future directions include expanding to additional KP isolates with various FOF phenotypes.

**Disclosures:**

**Elizabeth B. Hirsch, PharmD**, Melinta Therapuetics: Honoraria|Merck and Company, Inc.: Grant/Research Support

